# Emotional Cues during Simultaneous Face and Voice Processing: Electrophysiological Insights

**DOI:** 10.1371/journal.pone.0031001

**Published:** 2012-02-22

**Authors:** Taosheng Liu, Ana Pinheiro, Zhongxin Zhao, Paul G. Nestor, Robert W. McCarley, Margaret A. Niznikiewicz

**Affiliations:** 1 Department of Psychology, Second Military Medical University, Shanghai, China; 2 Clinical Neuroscience Division, Laboratory of Neuroscience, Department of Psychiatry, Boston VA Healthcare System, Brockton Division and Harvard Medical School, Brockton, Massachusetts, United States of America; 3 Neuropsychophysiology Laboratory, CiPsi, School of Psychology, University of Minho, Braga, Portugal; 4 Department of Neurology, Neuroscience Research Center of Changzheng Hospital, Second Military Medical University, Shanghai, China; 5 University of Massachusetts, Boston, Massachusetts, United States of America; Cuban Neuroscience Center, Cuba

## Abstract

Both facial expression and tone of voice represent key signals of emotional communication but their brain processing correlates remain unclear. Accordingly, we constructed a novel implicit emotion recognition task consisting of simultaneously presented human faces and voices with neutral, happy, and angry valence, within the context of recognizing monkey faces and voices task. To investigate the temporal unfolding of the processing of affective information from human face-voice pairings, we recorded event-related potentials (ERPs) to these audiovisual test stimuli in 18 normal healthy subjects; N100, P200, N250, P300 components were observed at electrodes in the frontal-central region, while P100, N170, P270 were observed at electrodes in the parietal-occipital region. Results indicated a significant audiovisual stimulus effect on the amplitudes and latencies of components in frontal-central (P200, P300, and N250) but not the parietal occipital region (P100, N170 and P270). Specifically, P200 and P300 amplitudes were more positive for emotional relative to neutral audiovisual stimuli, irrespective of valence, whereas N250 amplitude was more negative for neutral relative to emotional stimuli. No differentiation was observed between angry and happy conditions. The results suggest that the *general* effect of emotion on audiovisual processing can emerge as early as 200 msec (P200 peak latency) post stimulus onset, in spite of implicit affective processing task demands, and that such effect is mainly distributed in the frontal-central region.

## Introduction

The ability to extract emotional salience from visual and/or auditory signals has important implications for effective functioning in social environment. Due to their emotional significance, emotional stimuli are thought to capture attention automatically when compared with neutral stimuli, both in visual and auditory modalities [1]–[5].

Studies in the last decades shed light on the neural processes underpinning emotional processing. Most of the studies examined emotion processing from unisensory stimuli, such as voice (prosody processing) [3], [5], [6] and faces [1], [4], [7]. There were also some studies that examined the simultaneous processing of emotional visual and auditory signals [8], [9]. The use of event related potential (ERP) methodology for the study of emotional processing during face and/or voice is particularly advantageous since it affords tracking neurocognitive processes as they happen in real time from the millisecond the stimulus is presented and thus provides a window of inquiry into these processes before a response is made.

Studies on emotional face processing suggest that emotion modulates early processing stages and that the processing of emotional facial expressions is automatic, i.e. not dependent on directed attention [10]–[12]. The early effects of emotion on face processing point to a first differentiation between neutral and emotional signals but not between different types of emotion (from 110 to 250 ms, including P200 and N250 ERP components) [13], observed mainly at frontocentral electrodes [1]. The differentiation between specific emotions seems to occur at later stages, from 400 to 750 ms (including the Late Positive Potential-LPP component) [1]. Of note, the N250 has been proposed to index processes related to decoding of emotional content from faces [14], [15], in contrast to the N170 that has been related more consistently to the structural encoding of faces [11], [16], [17]. Some of these studies indicate that the analysis of stimulus emotional salience and the structural encoding of faces (as indexed by the N170 ERP component) are partially independent processes [1], [12], [13]. Finally, emotion effects were also reported for P300 amplitude in studies using face stimuli [18]–[20].

ERP studies on auditory emotional processing (including stimuli such as speech prosody [3], [21], music [22], or environmental sounds [23] have reported early modulatory effects of emotion on auditory sensory and perceptual processing. These effects were observed on N100 and P200 amplitude, including more negative N100 amplitude for neutral relative to emotional vocalizations [2], more positive P200 for happy than neutral or sad voice stimuli [22], more positive P200 for neutral relative to emotional prosody [3], or more positive P200 for emotional relative to neutral vocalizations [2], [24]. These effects were more pronounced at frontocentral electrodes [2]. Together, these findings suggest that N100 may index the earliest point at which emotional and non-emotional vocalizations can be distinguished, likely along sensory, acoustic dimentions, and that P200 is an index of the extraction of emotional salience from acoustic cues whether or not they contain linguistic information. In addition, effects of emotional valence were also consistently reported for P300 amplitude [1], [25], [26].

In spite of advances observed in recent years [8], [9], [22], [27]–[33], there is still a dearth of data on how emotional information from one modality can influence processing in the other modality and how these two sources of information interact. Studies using audiovisual stimuli suggest that during affective processing inputs from one modality influence perception in another modality. Behavioral studies of audiovisual integration indicate that two congruent stimulus modalities may improve processing, as indexed by shorter reaction times and higher accuracy, when compared to unimodal stimulus [34]–[36]. The few ERP studies on multisensory integration of emotional information argue for an early (in the first 225 msec) integration of non-verbal emotional information from visual (face or picture) and auditory (voice or music) modalities. Reductions in the amplitude and latency of components of interest have been taken as evidence of the influence of one modality on the other, although enhanced amplitude and longer latencies have been also reported. Audiovisual integration was found to operate at the level of pre-attentive processes as indicated by mismatch negativity (MMN) study results [31], suggesting that the integration of emotional information from face and voice occurs automatically and before both have been fully processed independently. In addition, the evidence of early integration of emotional information was found at the level of N100, as reflected by its increased amplitude when the emotion in face and voice was congruent relative to when the emotion was incongruent [32]; this congruence effect did not occur when faces were presented upside-down. Giard and Peronnet [37], investigating multisensory integration of auditory and visual information from simple objects, found that audiovisual processing was associated with reduced early sensory components relative to the visual modality. Similar effects of increased processing speed and reduced effort in processing multisensory relative to unisensory non-emotional face and voice stimuli were reported by Brefczynski-Lewis and colleagues [38]. Of particular note, in the study of Pourtois et al. [33] that investigated the integration of congruent (happy and fearful) facial expressions and prosody, an earlier posterior P2b component was observed for congruent face-voice trials relative to incongruent trials, indicating delayed auditory processing for incongruent facial contexts. These findings suggested that the cross-modal integration of emotion occurs at early (perceptual) stages during auditory processing. Finally, Spreckelmeyer et al. [22] testing the combined perception of emotional cues (happy and sad vs. neutral) via the simultaneous presentation of matched or mismatched affective pictures (from the International Affective Pictures System - IAPS database - [39]) and affective sung notes, found a modulation of P200 and late positive potential (LPP) components. In particular, congruent happy pictures and voices led to more positive P200 amplitude in an attended relative to unattended condition, and congruent sad pictures and voices led to more positive-going late-positive potential (LPP).

While the Spreckelmeyer et al. [22] and Pourtois et al. [33] studies clearly suggested interactions between auditory and visual channels in terms of modulating emotional and perceptual processes, their interpretation is difficult given the small sample size in these studies and a lack of a neutral condition in Pourtois et al. [33] that would allow a comparison between audiovisual effects in the presence and absence of emotional cues. Together, findings from unisensory and multimodal studies indicate early emotion effects (indexed by N100, P200, N250, and P300), suggesting that the detection of emotional signals is relatively automatic. However, it is still unclear how emotional cues presented in different sensory modalities (e.g., face and voice) are processed when presented simultaneously and what neural processes underpin these cognitive and perceptual events. To the best of knowledge, there are no published ERP studies that examine simultaneous emotion processing from (static) face and voice. Puce et al. [40] examined species specific congruency (using dynamic face and voice stimuli) effects, but the study focused on the flexible use of emotionally neutral information rather than on the processing of emotional information from multiple channels.

The current study aimed at investigating electrophysiological correlates of processing neutral vs. emotional (angry and happy) cues in simultaneously presented static faces and non-semantic sounds. Non-semantic sounds were chosen to avoid the confounding effects of linguistic (e.g., semantic) information on emotion processing. An additional motivation to use non-semantic emotional sounds was the fact that they constitute an important vehicle to convey emotional attitudes and mood states.

In addition, we were interested in examining the effects of emotional processing of audiovisual stimuli in the absence of task requirements that would focus on these emotional cues. Given the significance of emotion for survival, and the fact that in daily life emotion is conveyed from multisensory modalities in an automatic way and often without full conscious awareness [41], we expected that this design would provide us with a more ecologic account of multimodal emotional processing from auditory and visual modalities. In order to maintain attention to the task, participants were instructed to detect a monkey face from a series of faces that contained either neutral or emotional expressions.

We hypothesized that, consistent with previous studies on affective multimodal integration, we would observe a differentiation between neutral and emotional conditions in early components, particularly before 300 msec. Given the sensitivity of N100 and P200 amplitude to attention, and the relevance of emotional information to survival, we expected that the emotional conditions would capture more attentional resources than the neutral condition, which would be indexed by enhanced N100 and P200 amplitude. In addition, we hypothesized that later components would be also sensitive to emotional manipulations, in particular the N250 and P300 [1], [14], [15], [25], [26].

Based on previous accounts of increased positivity for emotional relative to neutral stimuli in the 300 msec latency window that spans both the N250 and P300 components [42], we expected reduced N250 and increased P300 for emotional relative to neutral stimuli. Given inconsistent findings related to specific *emotion* effects on the P300 component [10], [42], [43], we aimed at investigating whether P300 recorded to complex emotional audiovisual input distinguishes between emotional and neutral information, similarly to earlier components, or whether it distinguishes between specific emotional categories, here happy and angry faces. We reasoned that audiovisual emotion processing effects will be observed at fronto-central location and that parieto-occipital components associated with structural aspects of face processing (including N170) will not be sensitive to emotion processing effects, as suggested by previous studies [1], [12], [13].

## Results

### 1. Behavioral data

The mean response time to targets was 702.98±192.05 ms with 97.94% response accuracy. The high response accuracy suggested that subjects stayed engaged by the task and that their attention was appropriately maintained.

### 2. ERP data

**Figure 1:**
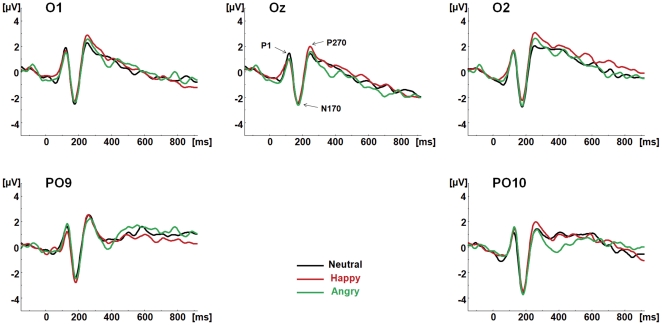
ERP waveforms to each condition at parieto-occipital electrodes. The parieto-occipital components P1, N170 and P270 are shown at O1, Oz, O2, PO9 and PO10 for the neutral (Black), happy (Red) and angry (Green) congruent audiovisual conditions (face and voice).

**Figure 2:**
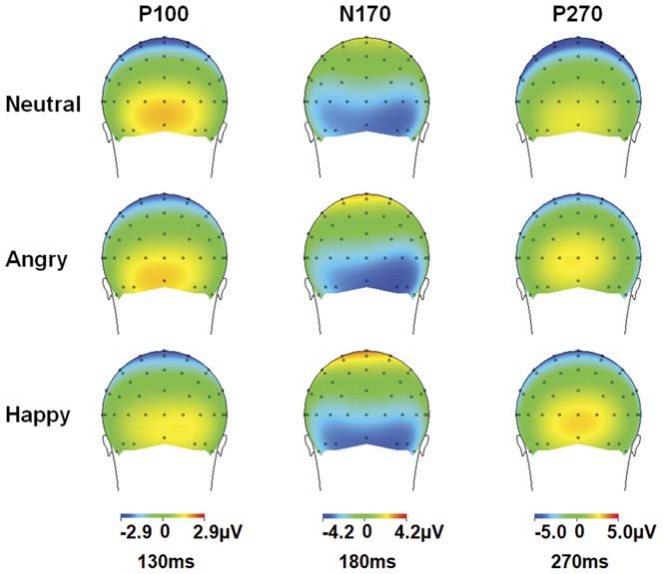
Scalp topographies for the parieto-occipital ERPs components. The voltage topographies of the parieto-occipital components on the scalp are shown for Neutral (top row), Angry (middle row) and Happy (bottom row) congruent audiovisual conditions (face and voice) at 130, 180, and 270 ms.

Figure 1 shows grand average waveforms for neutral and emotional conditions, at parieto-occipital electrodes. Figure 2 illustrates the topographic distribution of parieto-occipital components for each condition.

### 2.1. Parieto-occipital components

#### P100

No significant main effect or interaction involving the factor *condition* was found for P100 amplitude or latency (*p*>0.05).

#### N170

As for P100, there was no significant main effect or interaction involving the factor *condition* (*p*>0.05).

#### P270

No significant main effect or interaction involving *condition* factor was found for P270 (*p*>0.05). The lack of condition differences for all parietal-occipital components was retained when the data were re-analyzed using the average reference.

### 2.2. Frontal-central components

**Figure 3:**
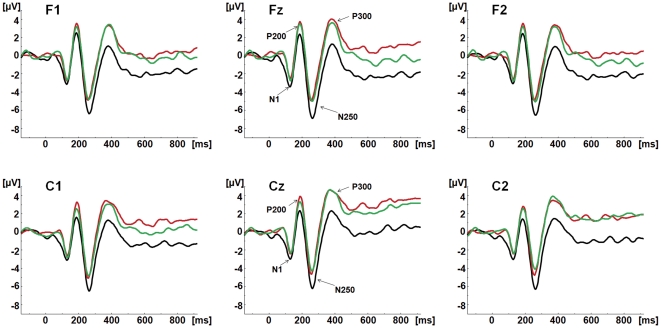
ERP waveforms to each condition at fronto-central electrodes. The fronto-central components N1, P200, N250 and P300 are shown at F1, Fz, F2, C1, Cz and C2 for the neutral (Black), happy (Red) and angry (Green) congruent audiovisual conditions (face and voice).

**Figure 4:**
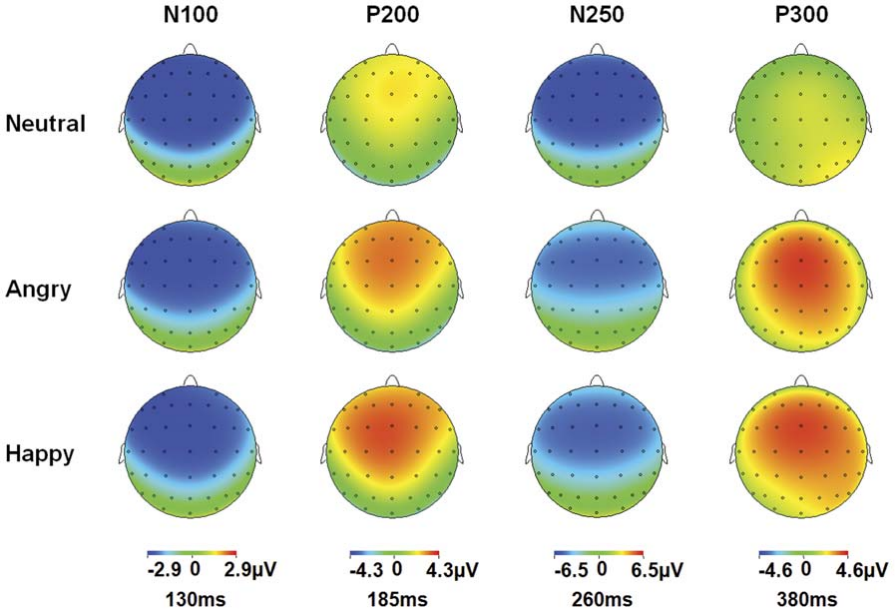
Scalp topographies for the fronto-central ERPs components. The voltage topographies of the fronto-central components on the scalp are shown for Neutral (top row), Angry (middle row) and Happy (bottom row) congruent audiovisual conditions (face and voice) at 130, 185, 260 and 380 ms.

Figure 3 shows grand average waveforms for neutral and emotional conditions, at fronto-central electrodes. Figure 4 illustrates the topographic distribution of fronto-central components for each condition.

#### N100

N100 amplitude to Happy and Angry condition seemed less negative relative to neutral condition. However, the statistical results showed no significant main effect or interaction involving condition factor (*p*>0.05).

#### P200

The MANOVA on P200 amplitude yielded a significant main effect of region (*F*(1,17) = 11.532, *p* = 0.003): P200 amplitude was more positive at frontal relative to central region. A significant main effect of condition (*F*(2,16) = 3.919, *p* = 0.041) was also observed: P200 was less positive for neutral (mean = 2.58 µV) relative to happy (p = 0.044; mean = 4.013 µV) condition, while neither neutral or happy condition was significantly different from Angry (mean = 3.57 µV) condition.

No significant main effects or interactions were observed for P200 latency.

#### N250

The MANOVA on N250 amplitude yielded a significant main effect of condition (*F*(2,16) = 4.934, *p* = 0.021): N250 was more negative for neutral (mean = −6.59 µV) relative to both Angry (*p* = 0.048; mean = −5.08 µV) and Happy (*p* = 0.033; mean = −5.15 µV) conditions, and no difference was found between Angry and Happy condition (*p* = 1.000).

The MANOVA on N250 latency yielded a significant main effect of condition (*F*(2,16) = 8.664, *p* = .003): N250 peaked earlier for Happy (mean = 257.50 msec) relative to Angry (*p* = 0.038; mean = 261.81 msec) and Neutral (*p* = 0.003; mean = 264.29 msec) conditions, with no difference between the two latter conditions.

**P300.** The MANOVA on P300 amplitude yielded a significant main effect of condition (*F*(2,16) = 7.697, *p* = 0.005): P300 was less positive for neutral (mean = 2.21 µV) than for both angry (*p* = 0.023; mean = 4.26 µV) and happy (*p* = 0.009; mean = 4.47 µV) conditions, with no difference between Angry and Happy condition (*p* = 1.000). No significant main effects or interactions were observed for P300 latency.

### 2.3. Correlation between P200 and N250

The relationship of P200 and N250 amplitude to each condition (Angry, Happy and Neutral) was examined using Pearson correlations at Cz. For Angry condition, there was a significant positive correlation between the P200 and N250 amplitudes (r = 0.557, *p* = 0.013); For Happy condition, this correlation was r = 0.520, *p* = 0.022; For Neutral condition, it was r = 0.762, *p* = 0.000. The results indicated that more positive P200 was associated with less negative N250.

## Discussion

This study aimed at investigating the processing of emotional cues during simultaneous presentation of face and voice. To this end, congruent faces and non-semantic sounds were presented, while subjects were asked to perform a visual categorization task, i.e., deciding if they saw a monkey face. Results showed that the extraction of emotional cues from multimodal input was associated with differential ERP activity at frontal-central sites, and was indexed by P200, N250, and P300. P100, N170 and P270 observed at parieto-occipital sites were not modulated by emotional content.

Fronto-central negativities showed a differential amplitude pattern: while N100 did not distinguish between neutral and emotional conditions, N250 was less negative to emotional relative to neutral face-voice pairings. Fronto-central positive components, P200 and P300 were more positive for emotional relative to neutral face-voice pairings. No component distinguished between the type of emotion presented (happy and angry). The emotion effects observed for P200, N250 and P300 suggest that emotional information cannot be ignored even when it is not task-relevant [48]. These results also suggest that emotion modulates both perceptual (P200, N250), and attentional (N250, P300) processes in the two sensory modalities.

In the following sections, we will separately discuss main findings for each component, integrating our findings with recent evidence on affective and multimodal processing.

### N100: the similar sensory processing of neutral and emotional audiovisual information

The audiovisual (face-voice) stimuli generated a robust N100 response; however, the N100 did not distinguish between the emotional types of stimuli.

N100 has been traditionally associated with sensory processes; it was found sensitive to physical characteristics of the stimuli and modulated by attention [49], [50]. In previous studies investigating multimodal integration of affective information, N100 was found sensitive to congruency effect: it was larger for congruent relative to incongruent angry face-voice pairs [32], which suggested an early integration of information coming from different inputs. However, that study did not use neutral condition.

In the current study congruency of inputs was not investigated but rather, we focused on implicit processing of emotion conveyed by auditory and visual modalities. The results of our study suggest that N100 does not provide a robust distinction between neutral and emotional audiovisual stimuli. Even though a distinction between neutral and emotional stimuli has been observed in previous studies with unimodal stimuli [51], this was not observed in our study with complex audiovisual input. Because of the sensitivity of N100 amplitude to attention, these results may suggest that both neutral and emotional audiovisual stimuli captured similar amount of attention at this early processing stage. Alternatively, given the implicit nature of emotion perception in this task, attention may not have played a major role in the early distinction between neutral and emotional information.

### P200: extraction of emotional salience and increased attention to relevant (emotional) audiovisual stimuli

The P200 was obtained to all stimuli and was the first component that indicated processing differences between emotional and non-emotional face-voice stimuli.

Traditionally, P200 has been associated with early categorization processes, is sensitive to physical features of stimuli and modulated by attention [52]–[55]. Studies on affective prosody processing have pointed to P200 sensitivity to extracting emotional salience from the auditory signal [3] and valence-specific orienting to relevant stimuli [56]. Studies on visual processing have also reported the relevance of P200 as an index of emotional salience detection [12], [13], [57]. Unimodal studies (both *visual* and *auditory*) suggest that the emotional differentiation irrespective of valence occurs around 200 msec after stimulus onset (e.g., *auditory* – [3]; *visual* – [12], [13]). In addition, this early differentiation may be independent of attention, occurring under implicit processing demands, as suggested by previous studies [32].

In the current study, increased P200 was found to emotional (both angry and happy) relative to neutral condition, even though the difference between angry and neutral condition did reach statistical significance. These results have been obtained independent of attentional focus. They suggest that the differentiation between emotional and non-emotional cues may happen outside of the focus of attention.

It is noteworthy that the differentiation between neutral and emotional stimuli but not between specific types of emotions at the level of P200 has been previously reported in the studies of prosody processing where electrophysiological correlates of emotional and neutral prosody were investigated [58]. In that study, P200 was proposed to index the extraction of emotional salience from the auditory signal. Thus, the results of the current study, in conjunction with the previous studies' results, suggest that the processes indexed by P200 and related to extracting emotional salience are not modality specific but rather operate on both auditory and audiovisual material [22]. This finding is consistent with the role of P200 in categorization processes that are likely not modality specific (differentiating if a stimulus is emotionally salient or not is a type of categorization).

### N250: decoding emotional cues for discrimination of audiovisual stimuli

While early components index more sensory aspects related to early stimulus categorization, later components are argued to index more complex cognitive and affect-related processes [56]. The fronto-central distributed N250 has been proposed to index early perceptual recognition processes and decoding of emotional cues (such as the extraction of emotional cues from faces [59]. Using a task of facial emotional recognition and perception of blurred faces and objects, Streit et al. [59] found a more pronounced N250 component elicited by the faces in the emotion recognition task, whereas this component was virtually absent to the faces in the blurred object condition. Similar results were also reported by Wynn et al. [60]: these authors used three tasks – a gender identification task, an emotion identification task, and a building identification task. The results indicated the largest N250 amplitude for emotion, followed by gender, and then by buildings. Unfortunately, both studies did not include a contrast between neutral and emotional faces.

In our study, less negative N250 amplitude was observed for emotional (both happy and angry) relative to neutral condition. These results are consistent with previous studies of emotional face processing reporting reduced amplitude of N250, VPP (P200) and P300 to emotional relative to neutral faces, irrespective of valence [12], [42].

Of note, in a study using dynamic audiovisual speech stimuli [61], a negative deflection peaking around 300 msec with a similar scalp distribution as the N250 in our study, was found to be more negative to incongruent than to congruent audiovisual speech stimuli. Also, another study showed that incongruous relative to congruous faces in a face identity-matching task elicited an enhanced negativity peaking around 350 msec with a frontocentral maximum [62]. This seems to indicate that difficulties in integration may be related to a more negative N250 amplitude. Thus, we may argue that our findings suggest that greater emotional salience of emotional audiovisual information leads to less difficulty in its processing. Consistent with this hypothesis, the N250 latency was earlier in the happy relative to neutral condition. Thus, while emotional audiovisual stimuli are related to enhanced stimulus elaboration (more positive P200), they also seem to be related to easier integration processes (less negative N250).

It is also possible that the two processes are closely related and influence each other such that the reduced N250 for emotional stimuli is due to the preceding enhancement of the P200 [63]. Of note here is the observation of a strong positive correlation between the anterior P200 and the N300 in an investigation of the differential processing of angry, happy, and neutral faces [56]. More positive P200 for emotional faces was associated with less negative N300 for the same stimuli. In our study, we observed a similar relationship between P200 and N250.

### P300: discrimination of emotional audiovisual information for enhanced elaboration and evaluation

Processes indexed by P300 are associated with processes such as stimulus recognition and discrimination [64]. In addition, the P300 has been shown to index the amount of attentional resources allocated to a stimulus [65]. Also, P300 amplitude can be modulated by valence: the reported *emotion effect* (i.e., more positive-going ERPs for emotional relative to neutral stimuli around 300 msec, including the P300 and N300 components – [66]–[68]) has been described mostly in visual studies.

Consistent with the role of P300 amplitude as an index of attention- and valence-oriented processes, the larger P300 amplitude for emotional relative to neutral condition found in our study suggests that emotional information has privileged access to processing resources [69], [70] and that participants use more attentional resources when integrating emotional audiovisual information than when integrating neutral audiovisual information. Also, the more positive-going amplitude for emotional stimuli is consistent with the sensitivity of P300 to stimulus valence, reported for pictures [71] and sounds [72].

Together, these results point to three major conclusions:

(1) Given the results of the current study, we conclude that even under implicit affective processing demands, emotion automatically attracts attention and modulates perceptual (P200, N250) and attentional (N250, P300) processes devoted to audiovisual stimuli.

(2) Given the results of the current study in conjunction with the results reported in the previous studies, we concluded that the processes indexed by N100, P200, N250, and P300 components do not seem to be modality specific but rather operate on sensory percepts and their higher order representations regardless of modality. This result suggests several possibilities: cross talk between visual and auditory modalities, the feedback loop from amodal regions of emotion processing back to primary visual and auditory cortices, and direct influence of frontally distributed regions including orbitofrontal and inferior frontal gyri devoted to higher order processing of emotional information on P200, N250, and P300.

The neural generators of these components are primarily confined to temporal structures [28], [63], so it is unlikely that they might reflect activity in the frontal regions devoted to emotion processing. Furthermore, it is believed that processes supported by the frontal regions are related to emotion identification and make distinction between specific emotions possible. Thus, the lack of a distinction between specific emotions for all the components discussed here speaks against the possibility that these higher order regions contributed to the scalp recorded amplitudes in a meaningful way.

Therefore, it is more likely that the current results reflect cross talk between visual and auditory modalities, the influence of early multimodal integration regions such as superior temporal sylcus on unimodal processing areas, or both. Several recent studies indicate direct communication between visual and auditory modalities as well as modulatory impact of early multimodal integration regions. For example, Saint-Amour et al., [73] reported the presence of McGurk MMN to mismatches between auditorily presented ‘ba’ and visually presented ‘va’. The first response was observed at 175 msec, followed by responses around 290 msec, and 350–400 msec that roughly correspond to effects observed in the current study in P200, N250 and P300 latency range. The effects in the Saint-Amour study were localized to the STG regions. Kayser et al. [74] reported the results of recordings from the auditory cortex in alert monkeys exposed to naturalistic audiovisual stimuli. The authors demonstrated that firing rates and timed spike patterns were more reliable when monkeys were watching congruent audiovisual events. Werner and Noppeney [75] using functional MRI approach showed three regions where evidence pointed to multisensory integration: primary auditory cortex, superior temporal and intraparietal sulcus, and ventrolateral prefrontal cortex. Functional connectivity analyses pointed to both direct contact between primary sensory cortices and feedback influences from the STS to primary cortices.

(3) Thus, the current results where distinction between neutral and emotional audiovisual facial expressions has been reflected across three components, P200, N250 and P300, suggest a sequence of processes of growing complexity with both feed-forward and feedback communication patterns between primary sensory regions and multimodal secondary areas. Presumably, the outputs of these processes will be analyzed further by prefrontal regions where emotional valence will be assigned. However, the ERP data obtained in this study did not reflect these processes. As suggested by Paulmann et al. [21], ERPs are not sensitive to aspects of emotion processing that are supported by frontal regions.

This study used an implicit task of emotion detection given its greater ecological validity. It is more frequent that people make judgments regarding emotional states of others within the context of other events rather than within the context of making explicit choices about emotions observed in other people. Nonetheless, it would be interesting to examine how, and to what extent, the processes observed here would change if the task of emotion identification was explicit rather than implicit. It would be also interesting to contrast congruent and incongruent emotional states expressed by faces and voices. Finally, we have tested only two emotions (angry and happy). More studies are needed to explore the differential processing of different emotion types presented through audiovisual channels.

### Conclusions

In this study, we presented evidence for early and late effects of the implicit processing of affective face-and- non-semantic vocalizations. Neutral and emotional (happy and angry) stimuli were differentiated at fronto-central electrode sites as reflected in P200, N250, and P300 components.

Neutral and emotional cues were not distinguished at the level of N100, suggesting a similar sensory processing of neutral and emotional information in audiovisual channels. More positive P200 amplitude for emotional relative to neutral condition indicated increased mobilization of attentional resources towards affective stimuli, regardless of valence. These findings suggested that P200 is an index of extraction of emotional salience from stimuli, allowing a first categorization between stimuli but not the discrimination between affective categories. The increased N250 for neutral relative to emotional stimuli indexed the decoding of emotional cues for subsequent discriminative processes, possibly also suggesting more difficult integration of neutral relative to emotional cues. Finally, the increased P300 for emotional relative to neutral condition suggested that both angry and happy cues provided in audiovisual input are related to enhanced processing and attentional resources, when compared with neutral stimuli, potentially related to their motivational value.

This study provided, for the first time, ERP evidence on the time course of implicit processing of emotional cues from concurrently presented faces and voices. This is particularly relevant for the understanding of social interactions where emotional signals are often processed in an implicit way and without full conscious awareness.

## Methods

### 1. Subjects

Twenty-two subjects participated in the study. Inclusion criteria included (a) right handedness (Edinburgh Inventory, [44]); (b) no history of neurological illness; (c) no history of alcohol or drug abuse; (d) no history of psychiatric disorder in oneself or in first-degree relatives; (e) no current medication for medical disorders that would have effects on electroencephalogram (EEG) morphology or consequences at the level or neurological and/or cognitive functioning; (f) verbal intelligence quotient (IQ) above 75; (g) no alcohol use in the 24 hours before testing; (h) no major sensory impairment that would prevent study participation (i.e., normal audition and normal or corrected vision); (i) an ability and desire to cooperate with the experimental procedures, as demonstrated by a signed given informed consent, following Harvard Medical School and Veterans Affairs Boston Healthcare System guidelines.

The subjects were recruited from local community by internet advertisements and paid for participation. Four participants were excluded from the sample due to excessive artifact in EEG data, leaving 18 subjects for subsequent data analyses. Table 1 shows the demographics characteristic of these subjects.

**Table 1:** Socio-demographic characterization of participants.

Variable	Value
Age (years)	44.61±7.84
Gender (M;F)	16;2
Education (years)	14.69±2.02
Subject's SES*	2.11±0.68
Parental SES	2.45±0.86
Verbal IQ**	105.39±11.28
Full Scale IQ	103.94±13.72

*SES = socioeconomic status.

**IQ = intelligence quotient.

This study has been approved by the Institutional Review Board of the Veterans Administration Boston Health Care System and by the Institutional Review Board of the Harvard Medical School. The approved informed consent form has been signed by all subjects.

### 2. Stimuli

All stimuli were audiovisual and consisted of the simultaneous presentation of a face and a voice. The experimental stimuli were human faces matched with a human voice based on emotion (angry, happy or neutral), gender (male and female) and age. Audiovisual controls were primate faces and species-specific vocalization.

The face stimuli that were a part of the audiovisual stimulus set included 108 human colored face photographs selected from the Radboud Faces Database [45] (www.rafd.nl). They consisted of 36 adult models (18 female) each displaying either an angry, happy or neutral facial expression (frontal gaze, 90°). All expressions were static. Pictures showed no facial hair, no glasses, makeup or jewelry. Face stimuli were equalized in brightness [45]. Mean valence of face emotional expressions was 2.03 (*SD* = 0.18) for angry faces, 4.27 (*SD* = 0.25) for happy faces, and 3.18 (*SD* = 0.33) for neutral faces (according to the validation scores published by Langner et al. [45]). Eleven primates faces (gorillas, macaques, and chimpanzees) were presented as controls. These were downloaded from free websites (image.Google.com; www.copyright-free-photos.org.uk). All faces had neutral expression and frontal gaze. Each photograph was trimmed so that only the face area was shown, and then adjusted to the same size (W×H: 326×425pixels) by using Adobe Photoshop CS4. All images were displayed centrally on a 20 inches CRT computer screen for 1500 msec at 7.4° vertical visual angle and 9.6° horizontal visual angle.

Auditory stimuli that were a part of the audiovisual stimulus set included 108 human and 7 primate digitized voices. The human voices were non-verbal vocalizations uttered by 9 male and 9 female individuals not participating in the experiment, and recorded through a microphone sitting on Sony MDR-7506 Modified Headset. The human voice set included 36 neutral, 36 happy and 36 angry sounds congruent with each of 3 emotional expressions in the human faces. For neutral sounds, 9 male and 9 female individuals were asked to make the sound ‘mmm’; for happy sounds, a fragment of a happy laugh was recorded when the individuals watched a funny movie clip; and for angry sounds, each individual was instructed to growl ‘humph’ angrily. Each individual was asked to produce 4 samples of each type of voice. Three judges were selected to assess the sounds. The two sounds of each category that were consensually rated by the three judges as the best representative of its category (neutral, happy, angry) were used in the study. All human voices were normalized (44 kHz, 16-bit, stereo, WAV-format) by using GoldWave v5.18, and adjusted to 72 dB in sound intensity and to 1500 msec duration. Mean fundamental frequency (F0) for angry voices was 246.30 Hz (*SD* = 64.80), for happy voices was 272.09 Hz (*SD* = 80.9), and for neutral voices was 168.35 Hz (*SD* = 49.3). A main effect of emotion was found for F0 (*F*(2,34) = 90.478, *p*<0.01): F0 was lower for neutral relative to both angry and happy voices (*p*<0.01). Subsequently, the same sounds were presented to 10 (3 females; mean age = 31.9±9.5) subjects who did not participate in the ERP experiment. These subjects were asked to assess the valence and arousal of each sound, by using a 9-point scale as in the Self-Assessment Manikin [46]. Mean valence ratings for angry sounds were 2.43 (*SD* = 1.11), for happy sounds were 7.07 (*SD* = 1.17), and for neutral sounds were 4.96 (*SD* = 0.48). Mean arousal ratings for angry sounds were 6.88 (*SD* = 1.12), for happy sounds were 5.89 (*SD* = 1.30), and for neutral sounds were 2.91 (*SD* = 1.51). In addition, participants were asked to indicate if each sound was “angry”, “happy”, “neutral”, or belonged to “other” emotional category. Angry sounds were judged as “angry” by 98.15% of participants, happy sounds were rated as “happy” by 100% of participants, and neutral sounds were rated as “neutral” by 98.15% of participants.

The auditory control stimuli that were a part of monkey face-voice stimulus set included primate vocalizations (coos and shrieks). Five of primate vocalizations were downloaded from the website http://www.monkeymania.co.uk/, and two were bought from http://www.soundsnap.com/.

For all sound files, the first and last 10 msec segments were faded out to avoid the on- and offset clicking transients. Auditory stimuli were presented binaurally through Sennheiser HD 380 PRO Headphones, at a comfortable sound level for each participant.

Each human face or voice was used one time; there were 27 monkey and 108 human audiovisual combinations. The monkey audiovisual stimuli served as response targets so that the processing of face-voice pairings would not be contaminated by a motor response, and the presence of a target would insure that subjects paid attention to each stimulus. The ERPs to monkey faces were not subjected to analysis. A total of one hundred and eight human audiovisual stimuli made up three emotionally *congruent* experimental conditions: angry (36), happy (36), and neutral (36).

All stimuli (one hundred eight face-voice parings and 27 monkey face-voice pairings) were presented in random order via SuperLab 4.2. The onset of (static) face and voice were synchronized to each other: both face and voice were presented at the same time and displayed for the same duration (1500 msec).

### 3. Procedure

**Figure 5:**
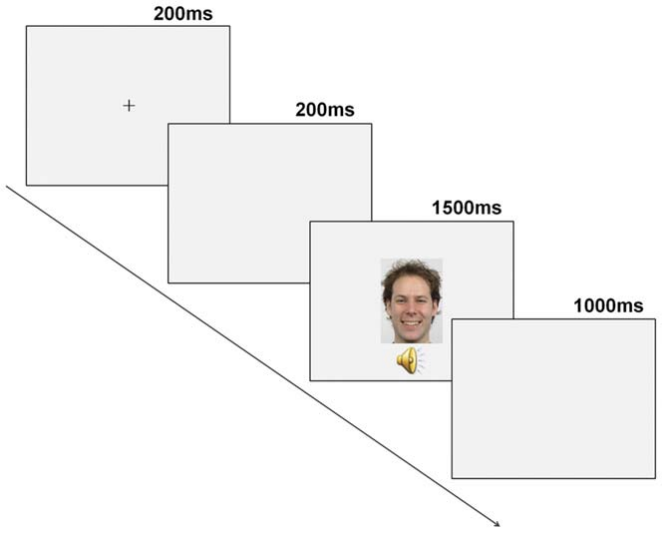
Schematic illustration of an experimental trial.

A trial started with a 200 msec fixation cross in the centre of the screen, followed by a 200 msec blank screen. Then, an audiovisual stimulus was presented for 1500 msec (face and voice presented simultaneously, with the same onset time), followed by a 1000 msec inter-stimulus interval (ISI) consisting of a blank screen (see Figure 5).

The experiment was conducted in a dimly-lit, sound-attenuated, and electrically shielded room. Subjects were seated 100 cm distance from the CRT computer screen and instructed to press a button using their right forefinger when they saw a monkey face.

### 4. EEG Recording and processing

Sixty-four channel EEG was collected using custom designed electrode caps from BioSemi system (Active 2). EEG was acquired in a continuous mode at a digitization rate of 512 Hz, with a bandpass of 0.01 to 100 Hz, and stored for later analysis. Blinks and eye movements were monitored via electrodes placed on left and right temples and one above the left eye. Electrode impedances were kept below 5 Kohms.

### 5. ERP Data Analysis

EEG data were processed offline using the BrainVision Analyzer package (Brain Products GmbH, Munich, Germany) and referenced off-line to the mean of the right and left mastoids.

Individual ERP epochs were constructed starting 150 msec pre-stimulus onset and ending 925 msec after the stimulus onset. Eye blink and movement artifacts were corrected by the method of Gratton, Coles, and Donchin (BrainVision Analyzer package) [47]. Trials containing excessive eye movements, blinks, muscle activity or amplifier blocking were rejected off-line before averaging (single-trial epochs with voltage exceeding +100/−100 µV were rejected from further analysis). Individual averages were only considered for further analysis if at least 70% of segments available for a given condition passed the artifact rejection (all the cases reported met this criterion). Separate averages were calculated for each condition (Angry, Happy and Neutral), after subtraction of the 150 msec pre-stimulus baseline.

The mean number of segments in individual ERP averages for Angry, Happy and Neutral condition was 27.4±6.82, 28.5±5.74, and 29.2±5.14, respectively, and a one-way ANOVA did not show differences between conditions (*p*>0.05).

Based on visual inspection of ERP waveforms, distinct positive and negative components were identified in two different regions; in the parieto-occipital region these were: P100, N170 and P270 (see Figure 2 and 3); in the frontal-central region, N100, P200, N250 and P300 were identified (see Figures 4 and 5).

The peak amplitude and latency were measured for each component. ERP components' amplitude and latency were measured as the most positive (for positive components) or the most negative (for negative components) data point in a specified latency window – at parieto-occipital region: P100: 80–180 msec; N170: 150–230 msec; P270: 220–330 msec; at fronto-central region: N100: 80–160 msec; P200: 150–240 msec; N250: 210–320 msec; P300: 310–450 msec.

Amplitude and latency of parieto-occipital components were separately subjected to multivariate analyses (MANOVAs) with condition (angry, happy and neutral) and electrode (O1/Oz/O2, PO9/PO10, PO7/PO8) as within-subject factors.

Amplitude and latency of fronto-central components were separately subjected to MANOVA analyses with region (frontal, central), condition (angry, happy and neutral) and electrode (Fz, F1/F2, F3/F4, Cz, C1/C2, C3/C4) as within-subject factors. Wilks' Lambda was used in all MANOVA analyses. Main effects were followed with planned comparisons with Bonferroni correction.

### 6. Comparison between different referencing schemes

In addition to the use of the mean of the right and left mastoids as a reference, we have also analyzed all data using the average reference to examine if the posterior electrodes in particular were affected by the choice of the reference (see Table S1 and Figure S1). As can be seen in Table S1 that compares the statistical results as a function of the reference, and from Figure S1, the results for the amplitudes of the components at posterior sites, which indeed were reduced by the use of the mean of the left and right mastoid reference, did not differ between the two referencing schemes. The amplitudes of the components at fronto-central sites were affected by the use of the average reference: they were reduced with the use of the average reference. Since our primary interest was in the centro-frontal sites given the evidence that these sites are sensitive to emotional manipulations, we report and discuss the results from the mean of the left and right mastoid referencing scheme.

## Supporting Information

Figure S1 The comparison of ERP waveforms obtained with the mean of left and right Mastoid-Reference (Mastoid Ref) and Average-Reference (Average Ref). Left: ERPs waveforms to each condition at Cz (top) and PO10 (bottom) created with the use of the mean of the right and left mastoids as a reference method; Right: ERPs waveforms to each condition at Cz (top) and PO19 (bottom) created with the use of common average as a reference method. Compared with Average-Ref, the use of Mastoid-Ref method yielded larger components (N1, P200, N250 and P300) at Cz, but smaller components (P1, N170 and P270) at PO10.
(TIFF)

Table S1 Comparison of the MANOVA results from Mastoid-Ref and Average-Ref (Mastoid-Ref: the use of the mean of the right and left mastoids as the reference; Average-Ref: the use of the average of the active channels as reference).
(DOC)
