# Supplementary figures and images for: Emotional Cues during Simultaneous Face and Voice Processing: Electrophysiological Insights
Source: PLoS One. 2012 Feb 22;7(2):e31001. doi: 10.1371/journal.pone.0031001 (PMC3285164; doi:10.1371/journal.pone.0031001)

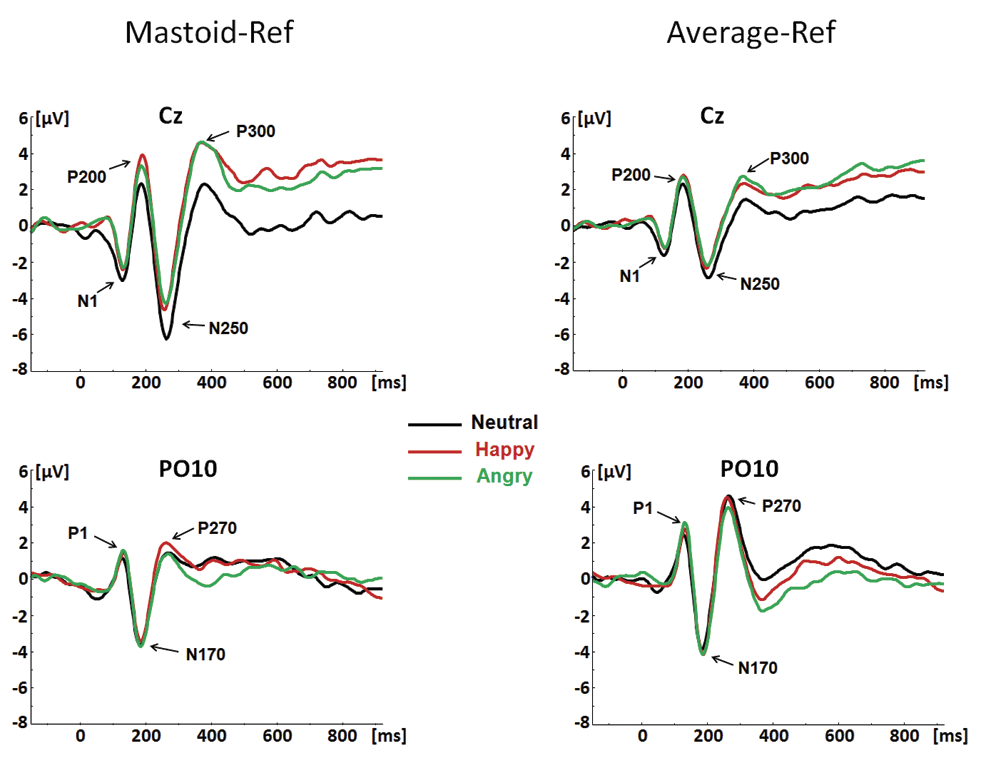

Supplement: Figure S1 — The comparison of ERP waveforms obtained with the mean of left and right Mastoid-Reference (Mastoid Ref) and Average-Reference (Average Ref). Left: ERPs waveforms to each condition at Cz (top) and PO10 (bottom) created with the use of the mean of the right and left mastoids as a reference method; Right: ERPs waveforms to each condition at Cz (top) and PO19 (bottom) created with the use of common average as a reference method. Compared with Average-Ref, the use of Mastoid-Ref method yielded larger components (N1, P200, N250 and P300) at Cz, but smaller components (P1, N170 and P270) at PO10. (TIFF) [file pone.0031001.s001.tiff]
