# Supplementary material for: Emotional Cues during Simultaneous Face and Voice Processing: Electrophysiological Insights
Source: PLoS One. 2012 Feb 22;7(2):e31001. doi: 10.1371/journal.pone.0031001 (PMC3285164; doi:10.1371/journal.pone.0031001)
Supplement: Table S1 — Comparison of the MANOVA results from Mastoid-Ref and Average-Ref (Mastoid-Ref: the use of the mean of the right and left mastoids as the reference; Average-Ref: the use of the average of the active channels as reference). (DOC) [file pone.0031001.s002.doc]

**Table S1.** Comparison of the MANOVA results from Mastoid-Ref and Average-Ref.

| **Component** | Condition effect | | | | Region effect | | | | Condition by Region | | | |
| --- | --- | --- | --- | --- | --- | --- | --- | --- | --- | --- | --- | --- |
|  | Mastoid-Ref | | Average-Ref | | Mastoid-Ref | | Average-Ref | | Mastoid-Ref | | Average-Ref | |
|  | F | Sig | F | Sig | F | Sig | F | Sig | F | Sig | F | Sig |
| **Frontal-central** |  |  |  |  |  |  |  |  |  |  |  |  |
| N100 Amplitude | 1.092 | 0.359 | 1.115 | 0.352 | 2.737 | 0.116 | 4.34 | 0.053 | 0.193 | 0.826 | 0.223 | 0.803 |
| N100 Latency | 1.259 | 0.311 | 1.818 | 0.194 | 0.002 | 0.965 | 0.00 | 0.997 | 3.201 | 0.068 | 2.393 | 0.123 |
| P200 Amplitude | 3.919 | **0.041** | 4.325 | **0.032** | 11.532 | **0.003** | 14.849 | **0.001** | 0.838 | 0.451 | 0.075 | 0.928 |
| P200 Latency | 2.384 | 0.124 | 2.045 | 0.162 | 1.151 | 0.298 | 0.185 | 0.672 | 0.812 | 0.461 | 1.545 | 0.243 |
| N250 Amplitude | 4.934 | **0.021** | 3.331 | 0.062 | 0.935 | 0.347 | 1.978 | 0.178 | 0.590 | 0.566 | 0.219 | 0.806 |
| N250 Latency | 8.664 | **0.003** | 8.017 | **0.004** | 0.001 | 0.970 | 0.180 | 0.676 | 1.614 | 0.230 | 0.894 | 0.428 |
| P300 Amplitude | 7.697 | **0.005** | 16.194 | **0.000** | 0.999 | 0.332 | 0.656 | 0.429 | 0.288 | 0.753 | 0.511 | 0.609 |
| P300 Latency | 0.938 | 0.412 | 0.258 | 0.776 | 0.104 | 0.751 | 1.105 | 0.308 | 0.580 | 0.571 | 2.066 | 0.159 |
|  |  |  |  |  |  |  |  |  |  |  |  |  |
| **Parietal-occipital** |  |  |  |  |  |  |  |  |  |  |  |  |
| P1 Amplitude | 0.050 | 0.951 | 2.708 | 0.097 |  |  |  |  |  |  |  |  |
| P1 Latency | 3.204 | 0.068 | 1.819 | 0.194 |  |  |  |  |  |  |  |  |
| N170 Amplitude | 0.178 | 0.838 | 3.340 | 0.061 |  |  |  |  |  |  |  |  |
| N170 Latency | 3.110 | 0.072 | 1.976 | 0.171 |  |  |  |  |  |  |  |  |
| P270 Amplitude | 2.275 | 0.135 | 0.609 | 0.556 |  |  |  |  |  |  |  |  |
| P270 Latency | 0.140 | 0.870 | 1.487 | 0.256 |  |  |  |  |  |  |  |  |
